# Aging and Interferons: Impacts on Inflammation and Viral Disease Outcomes

**DOI:** 10.3390/cells10030708

**Published:** 2021-03-23

**Authors:** Emily Feng, Elizabeth Balint, Sophie M. Poznanski, Ali A. Ashkar, Mark Loeb

**Affiliations:** Department of Medicine, McMaster University, Hamilton, ON L8S 4L8, Canada; fengey@mcmaster.ca (E.F.); balins1@mcmaster.ca (E.B.); poznans@mcmaster.ca (S.M.P.); loebm@mcmaster.ca (M.L.)

**Keywords:** type I IFN, type II IFN, type III IFN, aging, inflammation, immunopathology, monocyte/macrophages

## Abstract

As highlighted by the COVID-19 global pandemic, elderly individuals comprise the majority of cases of severe viral infection outcomes and death. A combined inability to control viral replication and exacerbated inflammatory immune activation in elderly patients causes irreparable immune-mediated tissue pathology in response to infection. Key to these responses are type I, II, and III interferons (IFNs), which are involved in inducing an antiviral response, as well as controlling and suppressing inflammation and immunopathology. IFNs support monocyte/macrophage-stimulated immune responses that clear infection and promote their immunosuppressive functions that prevent excess inflammation and immune-mediated pathology. The timing and magnitude of IFN responses to infection are critical towards their immunoregulatory functions and ability to prevent immunopathology. Aging is associated with multiple defects in the ability of macrophages and dendritic cells to produce IFNs in response to viral infection, leading to a dysregulation of inflammatory immune responses. Understanding the implications of aging on IFN-regulated inflammation will give critical insights on how to treat and prevent severe infection in vulnerable individuals. In this review, we describe the causes of impaired IFN production in aging, and the evidence to suggest that these impairments impact the regulation of the innate and adaptive immune response to infection, thereby causing disease pathology.

## 1. Introduction

Elderly individuals are known to be highly susceptible to viral infection and experience increased severity of infection and subsequent mortality compared to the younger population. Despite efforts to protect our elders, the critical question of why the elderly are at a greater risk of experiencing severe disease outcomes during infection is poorly understood. As we age, our ability to respond to viral infection is compromised through not only an inability to control the virus, but also through defects in the ability to regulate immune responses to infection. Severe infections in elderly individuals of both the severe acute respiratory syndrome coronavirus type 2 (SARS-CoV-2) and H1N1 influenza A virus (IAV) pandemic viruses have been defined by higher viral load, impaired viral clearance, and heightened inflammation and production of inflammatory cytokines [1,2,3]. 

One of the key regulators of inflammation and immune-mediated pathology is the interferon (IFN) response. The IFN response, including type I, II, and III IFNs, plays a critical role in antiviral defense through its involvement in directly preventing viral replication, while also activating and regulating the immune response [4]. In particular, IFNs regulate the immune-stimulatory and immune-suppressive functions of monocytes/macrophages and dendritic cells (DCs), which are critical in modulating viral disease outcomes. These include their regulation of neutrophil, natural killer (NK) cell, T cell, and B cell responses to promote viral clearance but prevent immune-mediated pathology. Various accounts have suggested that aging impairs early IFN production and signaling following infection through both a reduction in IFN-producing macrophages and DCs, and impairments in the pathways that induce IFN production [5,6,7,8]. Given the central role of IFNs in the antiviral response, the impairments in the IFN response in elderly individuals are likely a critical driver of poor disease outcomes through a lack of viral control and the defective regulation of immunopathology. In this review, we consider how a potent IFN response to infection is critical to preventing excessive inflammation and promoting tolerance during infection. We will assess how type I IFN influences monocyte/macrophage-regulated innate and adaptive immune responses to infection. Finally, we will consider the evidence describing the impact of aging on these responses and how they may be responsible for the dysregulation of immune responses to viral infection and the inability to prevent severe disease outcomes. 

## 2. The Paradoxical Role of Type I IFNs in Inflammation and Disease Tolerance

Type I IFNs play a pivotal role in not only controlling viral infection, but also in regulating inflammation that is implicated in both preventing and inducing immunopathology. Type I IFNs consist of a group of cytokines, most notably IFN-α and its multiple subtypes, and IFN-β, but also IFN-ε, IFN-κ, IFN-δ, IFN-τ, and IFN-ω [9]. Type I IFNs signal through the dimeric interferon-α/β receptor (IFNAR1) present on the majority of cells, both hematopoietic and non-hematopoietic, and initiate signaling canonically through the classical JAK-STAT signal pathway [9]. Activation of TYK2 and JAK1 leads to the phosphorylation of STAT1 and STAT2, and their heterodimerization, which forms a complex with the interferon response factor adaptor protein 9 (IRF9) [9,10]. This complex will translocate to the nucleus to bind to IFN-stimulated response elements (ISRE) to induce the expression of various IFN-stimulated genes (ISGs) [9]. 

The ability to induce a potent and early IFN response to infection consistently correlates with improved disease outcomes. Mouse models of viral infections demonstrate that a loss in type I IFN signaling not only increases viral replication, but also immune-mediated pathology [11,12,13]. Specifically, there is increasing evidence that the production of type I IFNs correlates with the disease severity of SARS-CoV-2 infections. Hadjadj et al. demonstrated that in the peripheral blood mononuclear cells (PBMCs) of SARS-CoV-2 infected individuals, patients with a more severe illness displayed reduced IFN-α production and ISG expression at time of symptom onset [14]. Individuals with genetic defects in IFN signaling also comprise 3.5% of severe COVID-19 patients [15]. Conversely, Lucas et al. did not observe appreciable differences in IFN-α shortly after disease onset; however, IFN-α levels correlated with a reduced viral load and a more rapid recovery period in patients with moderate disease [16].

The timing of the type I IFN response during infection has emerged as a key factor for inducing protective versus pathological inflammation. A retrospective study of COVID-19 patients found that early administration of IFN-α2b treatment following hospital admission drastically improved outcomes, while late administration increased mortality rates compared to untreated patients [17]. Furthermore, studies investigating the prognosis in Ebola virus (EBOV) infection found that a potent and early type I IFN signature and its subsequent immune activation is indicative of greater survival outcomes in humans [18,19,20]. Viruses can also inhibit elements of the IFN signaling pathway in order to promote their replication and survival. For instance, SARS-CoV-1, SARS-CoV-2, and Middle Eastern respiratory syndrome coronaviruses (MERS-CoV) are known to impair early IFN signaling events in mice [21,22,23,24]. Specifically, SARS-CoV-2 targets the phosphorylation of IRF3 [25]. In a SARS-CoV-1 mouse infection model, a delayed type I IFN allowed for uncontrolled viral replication and type I IFN-mediated immunopathology; however, early intervention with recombinant IFN-β up to 12 h following infection with SARS-CoV-1 assisted in the control of viral titer [21,22]. In another study, a strong but delayed type I IFN induction over 4 days in response to IAV infection in mice was pathogenic compared to mice with an earlier but blunted IFN response [26]. From these studies, it has become apparent that an early IFN signature during viral infection protects against severe inflammation, while delayed and dysregulated type I IFN production can conversely instigate or exacerbate pathology. Thus, the ability to mount an early type I IFN response is critical to reducing pathology through both controlling viral replication and regulating the immune response. Therefore, it is likely that delays or impairments in the ability to quickly induce type I IFNs during the process of aging underlies exacerbated disease during infection.

As mentioned, the role of type I IFNs in promoting recovery from disease lies in its ability to not only control viral replication, but also in negatively regulating immune responses to prevent immune-mediated damage. Type I IFNs are implicated in inducing NK cell function, while also suppressing both neutrophil and type II innate lymphoid cell (ILC2)-mediated pathology to mucosal viral infections [11,12,27]. The important functions of type I IFNs in regulating immune responses to infection will be further investigated during the course of this review.

## 3. Induction and Regulation of Type I IFN Responses to Viral Infection

**Figure 1:**
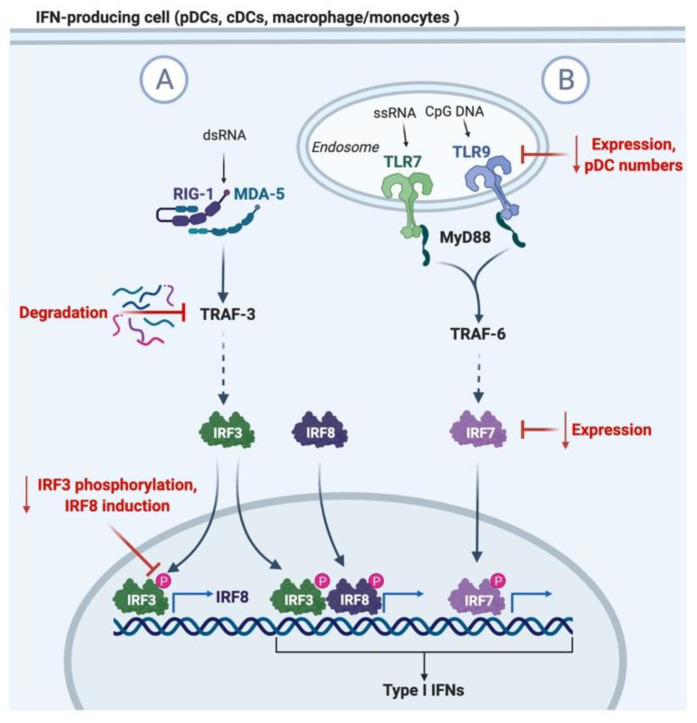
Diagram of type I IFN induction and mechanisms of impaired induction in aging. (**A**) Recognition of viral dsRNA in the cytosol by RIG-1 or MDA-5 leads to IRF3 activation of TRAF-3. Aging is associated with the degradation of TRAF-3 and the reduced phosphorylation of IRF3. IRF3 mediates the transcription of type I IFNs and IRF8, which feedbacks to amplify the expression of type I IFNs. (**B**) TLR7 and 9 recognition of viral ssRNA and CpG DNA in pDCs induces the activation of MyD88 and TRAF6, leading to the activation of IRF5 and 7, and the translocation to the nucleus for the transcription of type I IFNs. Aging leads to the reduction in pDC numbers in circulation, TLR7/9 expression, and IRF7 adaptor expression.

Type I IFNs are largely induced upon recognition of viral components through intracellular RIG1/MDA-5 receptors and endosomal-bound Toll-like receptors (TLR) TLR3, TLR7 (and TLR8 in humans), and TLR9 [10,28], as summarized in Figure 1. TLR7 and 9 signaling will induce type I IFN expression through the MyD88 and TRAF6 adaptors, and the activation of IRF7 transcription factor [28]. In addition, surface TLRs typically associated with the recognition of bacterial components can also induce type I IFN production in response to viral infections, such as TLR2 signaling in the herpes simplex virus (HSV), hepatitis C virus (HCV), measles virus (MV), and vaccinia virus (VV) infections [29,30]. Plasmacytoid DCs (pDCs) are a small subset of cells that are known for their robust ability to produce type I IFNs upon stimulation explicitly through TLR7 and TLR9 [31]. These cells are distinguished from classical DCs (cDCs) through their expression of markers such as B220 (CD45R), PDCA-1 (BST-2), BDCA-2, and BDCA-2, as well as their high expression of TLR9 [32].

In response to systemic infections with murine cytomegalovirus (MCMV), lymphocytic cytomegalovirus (LCMV), murine hepatitis virus (MHV), and HSV-1 and -2, pDCs provide a critical source of type I IFNs at early timepoints of infection [33,34,35]. pDCs have also been associated with IFN production in respiratory SARS-CoV-1 infection in mice [21]. Type I IFN signaling negatively regulates pDCs through the upregulation of pro-apoptotic genes such as Bim, Bid, Noxa, and Bax, and concurrently negatively regulate the expression of Bxl-xl and Bcl-2, thus limiting pDC type I IFN production in the early stages of infection [33,36,37]. This highlights the important function of type I IFNs at early time points of the immune response. Depletion of pDCs in genetic DTR-BDCA-1 mice only reduced IFN-β production at 36 h following MCMV infection [38]. In addition, antibody-mediated depletion of pDCs reduced IFN-α production up to 48 h following MHV infection and was sufficient to impair the control of viral replication and subsequent immune responses [38,39]. Likewise, TLR9^-/-^ mice, deficient in pDC production of type I IFNs, produce negligible levels of IFN-α in their serum at 6 h following systemic HSV-2 infection compared to wild-type mice [40]. Interestingly, recent evidence has suggested a correlation between reduced pDC levels in the peripheral blood with the severity of infection in COVID-19 patients [14].

In response to viral infection, and in particular during local mucosal infections, various other immune cells such as cDCs, inflammatory monocytes (IM), and macrophages are capable of producing robust levels of type I IFNs [29,34,41,42]. IM can produce type I IFNs during some viral infections through TLR2 signaling [29]. Meanwhile, alveolar macrophages (AM) are the major producers of type I IFN during both respiratory syncytial virus (RSV) and Newcastle disease virus infections in the respiratory tract [41,43]. Instead of recognizing viral pathogens with TLR7/8, cDCs and macrophages recognize and produce IFNs in response to pathogens through the RIG1/MDA5 pathway [44]. In the absence of AMs, pDCs were able to compensate for defective type I IFN production during respiratory infection, but did not offer the same level of protection [45].

## 4. Mechanisms of Impaired Type I IFN Signaling in Aging

There are many contributing factors towards a reduction in type I IFN production during aging, which involve both defects in signaling pathways and changes in pDC numbers and function. These defects are summarized in Figure 1. While the pool of cDCs are maintained with age, circulating pDCs decline, as do their ability to produce type I IFNs following in vitro stimulation [7,8,46,47,48,49]. These impairments are further exacerbated in elderly populations diagnosed with other co-morbidities that are risk factors for severe infection outcomes [8]. The reduced capacity of pDCs to produce type I IFNs with age is also partly due to reduced TLR7 expression in mice, and both TLR7 and TLR9 expression in human pDCs [8,47]. While the expression of the majority of components of the TLR signaling pathway remain unchanged with age, defects in IRF7 adaptor expression and translocation to the nucleus also underlie the impaired ability to stimulate type I IFN production [50]. These impairments are associated with increased reactive oxygen species and cellular damage seen in aging cells [50].

Impairments in the RIG1/MDA5 signaling pathway in aging also impact type I IFN production by cell types such as cDCs, monocytes, and alveolar macrophages. Secondary RIG-1 signaling serves to amplify IFN production through IRF8 transcription that stabilizes transcriptional machinery to the IFN promoters [51,52]. Molony et al. observed an increased targeting of the TRAF3 adaptor for proteasomal degradation during primary RIG-1 signaling in human monocytes, resulting in reduced IRF3 activation and IRF8 expression, thereby causing impaired amplification of IFN responses [5]. Therefore, evidence suggests that multiple sources of type I IFNs during viral infection are impacted during aging, and these deficits will not only impede viral control, but also impact immune activation and the control of immune-mediated pathology.

Increased rates of disease development in elderly individuals additionally contribute to deficits in their innate type I IFN response and susceptibility to severe viral infection. For instance, while IFN-producing pDCs undergo typically undergo contraction in numbers following early IFN production, type I IFN signaling during chronic infections maintains of a pool of exhausted pDCs with reduced type I IFN producing capacities, while depleting pDC progenitors in the bone marrow [53]. The combination of depleted progenitors and exhausted pDCs results in an impaired ability to react and mount a potent innate immune response to secondary infections [53,54]. Cytomegalovirus (CMV) has been specifically highlighted as a major contributor to immune exhaustion in elderly individuals [55,56,57]. Consequently, elderly individuals afflicted with chronic infections may experience increased susceptibility to infections through reduced type I IFN production capabilities, and as a result, have impaired viral control and immune induction. Likewise, obesity and its associated chronic inflammation correlates with impairments in the ability to mount an IFN response, and an increased susceptibility to IAV infection in mice [58,59]. Teran-Cabanillas et al. also demonstrated that PBMCs from obese individuals elicited reduced production of IFN-α and IFN-β to in vitro stimulation [60]. Thus, suppression of the type I IFN response by age-related co-morbidities likely plays a key role in worsening viral infection outcomes in the elderly.

## 5. The Role of Interferon and Aging in Regulating the Innate Immune Response

**Figure 2:**
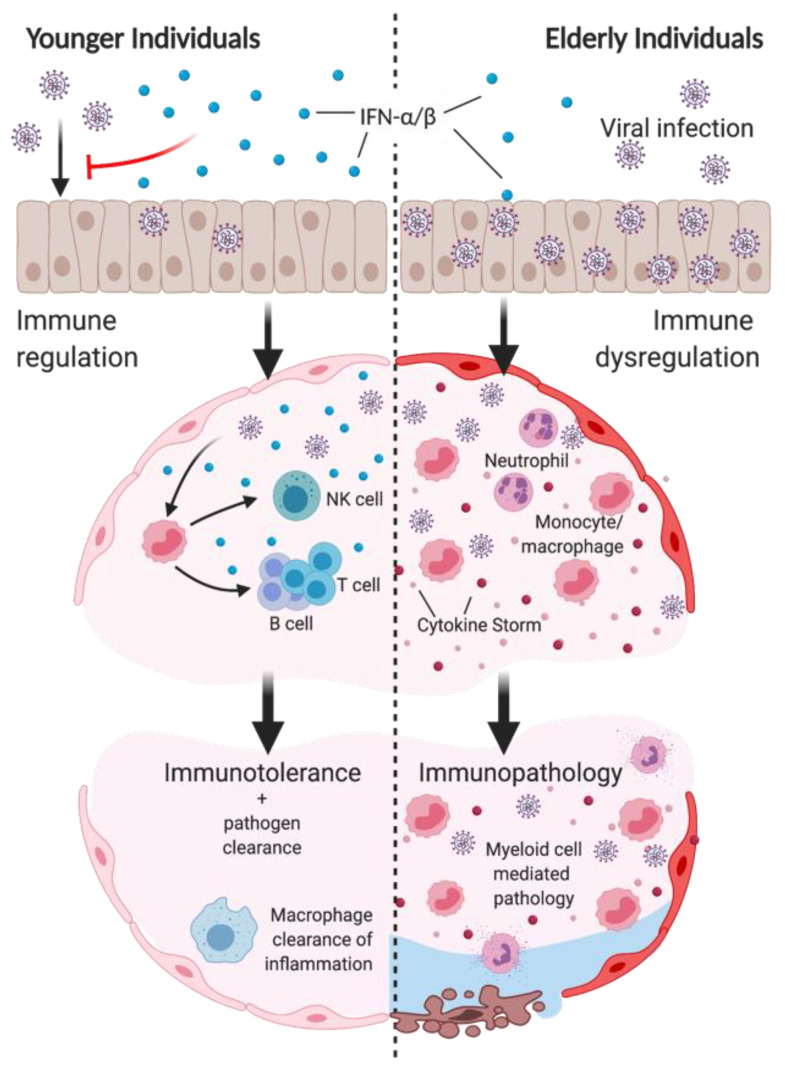
Impaired type I IFN induction in aging impairs viral clearance and promotes immunopathology following infection. Top: reduced IFN-α/β production in elderly individuals in the upper respiratory tract impair control of early viral replication. Middle left: type I IFN production of epithelial cells and myeloid cells regulates immune response to infection to promote viral clearance. Type I IFN responses induce inflammatory monocyte/macrophage (IMM) recruitment and activation, subsequently inducing NK cell recruitment and activation. IMM production of IL-12/15/18 promotes DC maturation to induce T and B cell functions. Bottom left: immune-mediated viral clearance, as well as macrophage-mediated resolution of inflammation promotes immune tolerance to infection. Middle right: Suppressed type I IFN induction in elderly individuals impairs the control of excessive IMM and neutrophil infiltration, and cytokine storm. Bottom right: IMM and neutrophils mediate significant damage to the airways and cause disease pathology to infection. Created with BioRender.com.

Impaired IFN response in the elderly has vast consequences on the induction and regulation of the innate immune response. As summarized in Figure 2, improper induction of IFNs not only impairs early viral clearance, but also the regulation of innate-mediated inflammation and tissue damage. A table comparing the impacts on the innate immune response by the loss of type I IFN or changes seen during aging can be found in Table 1.

### 5.1. Interferons Prevent Neutrophil-Mediated Inflammation

Neutrophils can protect against myxoma virus (MXYV) and IAV infection through their phagocytic functions and ability to unleash an arsenal of antimicrobial peptides through their formation of neutrophil extracellular traps [84,85]. However, their dysregulation during viral infection has been repeatedly shown to induce significant tissue damage in both liver and lung infections [11,62,86]. Excessive neutrophil recruitment is seen in the lungs of patients diagnosed with either severe IAV infection or severe SARS-CoV-2 [87,88,89]. Type I IFNs have been shown to suppress neutrophil-mediated pathologies in both chikungunya virus (CHIKV) and IAV infection in mice by regulating their recruitment to the site of infection [11,63,90,91]. Neutrophil recruitment in the absence of IFNAR was dependent on increased CXCL1 production by Ly6C^int^ monocytes in IAV infection and CXCL2 expression by monocytes in epicutaneous HSV-1 infection [11,64]. Meanwhile, CHIKV-infected mice had no changes in chemokine levels in the absence of IFN-β [90]. Nonetheless, it is clear that type I IFN signaling functions as a key regulator of neutrophil-mediated pathologies following viral infections.

Studies of aged mice have demonstrated an increase in neutrophil influx as a cause of heightened mortality following IAV and HSV-2 infection compared to young mice [61,62]. Heightened viral replication due to a deficit in type I IFN signaling in conjunction with increased IL-17 signaling can synergize to enhance neutrophil-mediated pathologies during infection [62]. Interestingly, Neupane et al. recently showed that IL-17A inhibited IFN-α2 production and the expression of ISGs during CHIKV infection by downregulating the expression of *Irf5* and *Irf7* in both structural and myeloid-derived cells, including bone marrow-derived macrophages and DCs [91]. Despite the reduction in the chemotactic ability of neutrophils seen in aged mice, these deficits are offset by the increased IL-17A production by aged NKT cells, as well as the increased production of the typical neutrophil-attracting chemokines, CXCL1 and CXCL2, in MCMV and HSV-2 systemic infection, resulting in liver injury [61,62]. In conclusion, the absence of type I IFN-mediated neutrophil regulation and virus control during aging could have drastic impacts on virus-induced pathology.

### 5.2. Macrophage Inflammatory and Anti-Inflammatory Functions Are Tightly Regulated by Type I IFNS

The regulation of both cytotoxic and anti-inflammatory populations of monocytes and macrophages remains essential for the clearance and tolerance of infection. Monocyte populations are classically characterized as IM or anti-inflammatory monocytes [92]. The recruitment of IM, defined as CCR2^+^Ly6C^hi^ in mice, is critical towards promoting viral clearance in a myriad of viral infections. Their roles include producing a plethora of cytokines, such as IL-12 and IL-18, to stimulate NK cell and TH1 activity, but they also display their own cytotoxic functions through producing nitric oxide [27,93,94,95]. The functions of IM in infection are strongly regulated by type I IFNs. The recruitment of IM is dependent on type I IFN induction of CCL2 in mucosal infection [11,27,41,68]. In the absence of IFNAR and impaired IM recruitment, anti-inflammatory monocytes demonstrate heightened inflammatory capacities and production of nitric oxide synthase 2 (NOS2) to potentially compensate for inflammatory outcomes [63]. However, these compensatory mechanisms may not be sufficient to control infection [63]. Likewise, proper type I IFN induction also serves to prevent excessive local IM proliferation as a mechanism to prevent IM-mediated pathology in IAV infection [69]. Type I IFNs have also been shown to suppress NOS2 production in IM during IAV infection, further demonstrating a critical function of type I IFNs in protecting against immunopathology [63]. Therefore, disturbances in later stage type I IFN production, such as during respiratory infections by alveolar macrophages, are associated with heightened inflammation and greater infection-induced pathology [69].

In SARS-CoV infection in mice, delayed early IFN signaling was associated with heightened viral load [21]. The type I IFN response at later time points of infection subsequently facilitated the excessive recruitment of pathogenic IMs, whose direct depletion potently suppressed pathology and mortality from infection [21,22]. Type I IFN signaling also induces the expression of TRAIL on macrophages, inducing alveolar epithelial cell apoptosis and consequently pneumonia in IAV infection [70,71]. These responses may improve the survival of infection, but have been largely associated with acute lung injury [70,71]. Thus, we observe a paradox, in which type I IFNs not only promote the induction, but also serve as a negative regulator of anti-viral IM function and IM-mediated pathology. Further investigation is needed to elucidate this careful balance of type I IFN function on IM activity. Overall, these findings suggest that disturbances in the balance and timing of IFN-regulated responses can result in impaired viral clearance and monocyte/macrophage mediated inflammation and tissue damage that underpin morbidity and mortality from infection. As both the ability to induce IFNs and the sensitivity to IFN signaling are impacted during aging, these changes may be sufficient to tip the scale towards a more inflammatory and damaging response.

The phagocytic function of macrophages is stimulated by type I IFN signaling, and the use of type I IFNs as a treatment not only improves phagocytic function, but also prevents infection-related mortality [66,73]. Absence of the expression of ISG15 in peritoneal F480^+^ macrophages impaired their phagocytosis of vaccinia virus (VACV)-infected cells in vitro, as well as their apoptosis [73]. Prostaglandin E2 (PGE2) expression induced by IAV infection inhibited type I IFN responses, thereby impairing monocyte and macrophage accumulation and apoptosis in the lungs [74]. Thus, promoting type I IFN induction and signaling enables macrophage phagocytic antiviral functions to control viral infection.

Phagocytic functions are also critical for the ability of alternatively activated macrophages to resolve inflammation. This includes the efferocytosis of apoptotic neutrophils, which, when not cleared, can result in secondary necrosis and inflammation [96,97]. Interestingly, the phagocytic function of macrophages can decrease during aging. In an IAV mouse model, aged mice displayed an impairment of macrophage-mediated efferocytosis of apoptotic neutrophils, resulting in increased inflammation and tissue damage during infection [66,98]. The inflammation resolution functions of macrophages are also regulated by type I IFNs. IFN-β-KO mice display impaired M2 polarization of monocyte-derived macrophages involved in the phagocytosis of apoptotic neutrophils following RSV infection [75]. During bacterial infection, IFΝ-β has also been shown to directly drive neutrophil apoptosis and uptake by phagocytic macrophages, as well as the downregulation of IL-6 and IL-12 while upregulating IL-10 [72]. IFN-α promotes the efferocytosis of apoptosis through the induction of tyrosine receptor kinase Axl in alveolar macrophages, thereby preventing secondary necrosis and subsequent inflammatory responses [99]. Thus, reduced IFN production in aging not only impairs IM function in the clearance of infection, but also the macrophage-mediated resolution of inflammation following infection.

### 5.3. Type I IFNs Are Essential for NK Cell Antiviral Functions

Type I IFN signaling has long been implicated in the activation and proliferation of NK cells, which play a central role in viral clearance. Type I IFN signaling promotes NK cell accumulation through inhibiting perforin-mediated fratricide amongst NK cells [82]. Furthermore, IM and DC production of IL-15 and IL-18 drives NK cell mobilization and effector functions [27,79,80]. The inhibition of IFN signaling, or the depletion of pDCs in mice, reduces nonspecific NK cell activation in response to viral infection and TLR agonists [38,81]. NK cells from aged mice displayed reduced cytotoxic function and IFN-γ production, both of which were dependent upon IFN signaling and correlated with impaired cDC IL-15 and IL-18 expression [76,77]. This suggests that defects in NK cell function in aging stem from impaired DC and IM activation by type I IFN. However, pDC-depleted mice infected with MCMV displayed an increased recruitment of MCMV-specific NK cells through the heightened production of IL-12 as a mechanism to enhance NK cell cytotoxic function to compensate for a lack of early antiviral control [38,100]. Type I IFN signaling on NK cells has also been shown to directly suppress NK cell IFN-γ production [101]. Thus, type I IFN modulates NK cell function in both positive and negative ways to promote viral clearance but prevent NK cell-mediated pathologies. While impaired early production of IFN-α/β in elderly individuals may significantly impact the early non-specific NK cell response to viral infection, the long-term impacts include an inability to clear viral infection and heightened immune activation that result in tissue damage.

## 6. Interferon, Aging, and the Adaptive Immune Response

Poor vaccine responses have been demonstrated in elderly populations when compared to the young [102,103,104]. A table comparing the loss of type I IFN to aging on the adaptive immune response to acute infection is summarized in Table 2. Here, we explore how defects in the innate immune response due to impaired type I IFN signaling in aging can result in impaired induction of a potent T and B cell adaptive immune response as summarized in Figure 2. Furthermore, we discuss how the ability to generate potent memory responses to vaccines or infection strongly relies on direct regulation by the type I IFN response [105,106,107,108].

### 6.1. Controlling the Balance between Protection and Destruction by T Cells

The magnitude of type I IFN signaling during early stages of infection is critical for regulating T cell responses to viral infections, thus any reductions caused by aging can disrupt this process [145]. It is evident that the induction of type I IFNs potently induces T cell responses. Absence of IFNAR or the depletion of pDCs in mice has been shown to impair viral clearance and correlates with a reduction in the maturation and function of antigen specific cytotoxic CD8^+^ T cells to HSV-1, MCMV, and VV and West Nile virus (WNV) [38,100,130]. Conversely, type I IFN signaling during chronic LCMV infection has been shown to induce PD-1 expression to suppress T cell activation, and impair de novo T cell priming and TH1 responses [146,147]. However, both blocking type I IFN-mediated negative regulation of T cell responses through neutralizing antibodies, or enhancing type I IFN signaling by administering recombinant IFN-α/β during LCMV infection allowed for viral clearance by preventing CD8^+^ T cell exhaustion and suppressing PD-1 expression [127,147,148,149]. Therefore, while insufficient type I IFN during chronic infection can suppress T cell function, a certain signaling threshold can be achieved for optimal T cell function that mediates viral clearance. By considering the impact of chronic infection on the levels of type I IFN production, these findings also highlight how chronic infections can further impair T cell responses in elderly individuals.

There are multiple mechanisms through which altered type I IFN signaling may impair T cell responses. The effects of reduced type I IFN signaling on early innate immune responses would inevitably impact T cell responses. For example, reduced IL-12, -15, and -18 production by IM and DCs due to a reduction in IFN production would contribute to reduced T cell activation [38,100,114,115]. Monocyte-derived DCs (moDCs) derived from IM in particular have been described as a robust source of IL-12 for the stimulation of TH1 cells [116], and type I IFN has also been shown to enhance moDC differentiation and activation in vitro [114,115]. Thus, the depletion of IM and consequently moDC severely impacted TH1 and CD8^+^ T cell responses to infection in mice [116,150]. The cross-priming of T cells by cDCs and moDCs also requires type I IFN signaling. Absence of IFNAR on cDCs, or pDC ablation, impaired TH1 responses to stimulation through impaired cDC maturation and upregulation of CD40, CD86, and MHC II [100,117,118,119,120].

Indirectly, apoptosis of infected macrophages provides a source of antigen for cDC priming, and compromised type I IFN responses reduce macrophage apoptosis to IAV and VACV infection [73,74]. Apart from their role in stimulating antigen presenting cell (APC) cross-priming, type I IFNs also function to promote T cell maturation and expansion of primed T cells, functioning as a “third signal” to stimulate T cell responses [128,129,151]. Virus-specific IFNAR-deficient CD8 T cells show reduced expansion in the spleen following acute LCMV infection and a reduced capacity to produce granzyme B [128]. Production of type I IFN also acts to maintain a pool of IL-2 to promote T cell activation and expansion [152].

There is evidence of impacts on impaired IFN signaling on T cell responses in aging studies. IAV infections in aged mice show reduced T cell expansion and cytotoxic function [121,122,123]. They have also been shown to express higher levels of PD-1, similar to mouse models of chronic infection with suppressed type I IFN production [113,127]. DCs isolated from aged mice exhibit reduced expression of costimulatory ligands and inflammatory cytokine production, as well as increased PD-L1/2 expression, inhibiting T cell priming and cytotoxic functions [109,110,111,112,113]. These impacts on DC maturation and the ability to produce inflammatory cytokine responses can be attributed to reduced type I IFN production. In conjunction with impaired IFN induction, impaired sensitivity to IFN signaling can drive deficits in the development of T cell responses. CD4 T cells isolated from elderly individuals display a reduced sensitivity to type I IFN signaling, resulting in reduced IL-2 production and the expression of the activation marker CD69 [125]. As type I IFN signaling has been shown to drive DC maturation and T cell activation, reduction in IFN production during aging as well as reduced sensitivity to IFN signaling could underlie reduced T cell responses to infection.

Elderly individuals display impaired memory CD4 T cell responses to infection and vaccination [105]. These changes are exacerbated by CMV infection when comparing seronegative elderly individuals, and young individuals [56], which may suggest a correlation with suppressed type I IFN production by chronic infection. Absence of type I IFN or the depletion of pDCs not only impairs T cell maturation and expansion, but also reduces T cell memory formation and cytotoxic function [79,128,132]. IL-15 and IL-18 production from IM in response to type I IFN promotes the expansion of T cells and the development of memory T cells following infection [79]. Reduction in numbers of moDC also impairs memory T cell responses [153]. Opposingly, the immunoregulatory functions of type I IFN suppress memory CD8^+^ T cells. Type I IFN induces the attrition of resident memory T cells during acute infection [133,134]. Memory CD8^+^ T cells are known to be capable of causing immunopathology towards heterologous infections, inciting T cell- and IFN-γ-mediated tissue damage [154]. Thus, attrition by type I IFNs may function to prevent the accumulation of resident memory T cell populations, their activation to unrelated antigens, and immunological space for new T cell responses. Goplen et al. revealed that chronic inflammation and fibrosis from IAV infection in aged mice was attributed to enhanced tissue resident CD8^+^ T cells in the lungs that persisted 60 days post-infection, highlighting the dangerous potential of bystander-activated memory T cells [126]. In conclusion, we see that type I IFNs play a dual role in promoting critical T cell responses to clear a viral infection, while also suppressing the pathogenic potential of memory T cells.

### 6.2. Promoting B Cell Responses and Memory to Infection

Vaccine responses in the elderly compared to younger populations show a drastic decrease in seroconversion [103,135]. These changes have been largely attributed to clonal expansion due to chronic infections that shrink the naïve B cell pool in elderly individuals, impairing the ability to recognize and form memories of new antigens [155]. B cell populations from elderly individuals also show impaired antibody production in vitro to IAV, as well as the reduced transcription of activation-induced cytidine deaminase (AID) [136,138]. This correlated with reduced isotype switching, and reduced IgG^+^IgA^+^CD27^+^ memory B cells, but there was no change in IgM memory B cells [136,138]. Splenic B cells in mice also displayed reduced proliferation to stimulation in vitro [156].

Interestingly, type I IFNs have been shown to play a significant role in enhancing B cell functions [106,107,108]. Not only does type I IFN signaling directly promote plasma cell differentiation, but it also enhances antibody production and isotype switching during viral infection [106,107,108]. In vitro stimulation of human PBMCs with TLR7 and TLR9 agonists, IAV, and rotavirus (RV) demonstrated pDC-dependent B cell proliferation and antibody secretion through type I IFN signaling [106,139,140,141]. DC-dependent antibody secretion in mice is also observed in response to RV infection and TLR3 agonist poly(IC) treatment [139,142]. Likewise, TLR7-mediated type I IFN production was shown to enhance B cell activation during systemic VSV infection in mice [107].

IL-12, -15, and -18 have been demonstrated to stimulate B cell maturation through B cell homing [157]. Thus, similar to T cells, impairments in type I IFN-dependent DCs and IM recruitment and activation would inevitably impact B cell responses to infection [11,27,114,115]. IL-6 signaling has been shown to synergize with IL-12 to promote naïve B cell activation [158]. Meanwhile, the recruitment of IL-6-producing DCs is dependent on the recruitment of NK cells by type I IFN [159].

TLR7 and TLR9 signaling induce T-bet transcription factor expression in B cells to induce IgG2a class switching, and the loss of T-bet expression reduced survival to murine gammaherpesvirus gHV68 [160,161]. T-bet expression also showed dependence on pDCs, as type I IFNs mediated a positive feedback loop on T-bet expression in murine B cells [143,144]. Interestingly, expression of T-bet in human B cells in response to TLR7 stimulation is significantly lower in elderly populations [137]. Thus, in conjunction with clonal expansion and the shrinkage of receptor diversity, reduced type I IFN production and IFN-mediated IM and cDC inflammatory cytokine production and T-bet expression drive impaired antibody responses and memory formation in response to infection and vaccination in elderly populations.

## 7. Type II Interferon Immunoregulatory Functions Are Dependent on Type I IFN Signaling

IFN-γ, the only type II IFN cytokine, signals through its IFN-γ receptor on a myriad of cells [9]. Similar to type I IFNs, IFN-γ is critical towards promoting the adaptive immune response through enhancing the expression of MHC I and II on APCs and the phagocytic functions of macrophages [162]. Interestingly, aged mice show transcriptional deficiencies in IFN-γ signaling and the induction of MHC II expression on macrophages [163]. Type II IFNs are produced by NK cells and T cells during viral infection [27,38,164]. The production of IFN-γ is strongly regulated by the type I IFN response. Thus, the impairments in type I IFN production and signaling during aging would inevitably impact IFN-γ production. NK cell antiviral responses are dependent on direct and indirect type I IFN signaling [82,165,166]. Further, defects in T cell activation by a loss or a reduction in type I IFN would also dampen IFN-γ production [38,100,130].

IFN-γ has also been found to have immunoregulatory functions. Moro et al. defined that IFN-γ was capable of suppressing pathology mediated by ILC2s and type II immunity, mainly through suppressing production of the type II cytokines IL-5 and IL-13 [12,167]. Administration of IFN-γ alone was capable of suppressing pathology to IL-33-induced airway hyper-reactivity by tissue-resident ILC2s [167]. ILC2s have also been show be critical in T and B cell responses through the IFN-γ-mediated recruitment of DC, and in the differentiation of moDCs [153,159,168]. Therefore, IFN-γ, like type I IFNs, demonstrate critical functions in the antiviral response by both promoting viral clearance and preventing immune-mediated pathology. However, whether or not impactful impairments in IFN-γ production or signaling occur during aging remains an important area to be investigated.

## 8. Type III Interferons Control Viral Replication at Mucosal Surfaces

Type III IFNs, a group of cytokines named IFN-λ1, IFN-λ2, IFN-λ3, and IFN-λ4, provide similar protection and activate similar signaling pathways to type I IFNs; however, they signal through their own IL28/IL29 receptors [169,170,171]. Type III IFN induction against viral infections is similarly dependent on both TLR and RIG1 signaling pathways [169,170], thus defects in the RIG1/MDA5 pathways involved in type I IFN induction in aged PBMCs also impair type III IFN production [5]. Aged human pDCs have been shown to secrete reduced levels of IFN-λ in response to CpG and IAV stimulation [172,173].

The key defining difference between type I and type III IFNs is that the type III IFN receptor is largely expressed in epithelial cells, and a small subset of immune cell populations such as pDCs and neutrophils [174,175,176,177,178]. Thus, the effects of type III IFNs are limited to these immune cell populations, and to mucosal tissues including the lung, stomach, intestinal lining, and reproductive tract, highlighting their critical role in defending against mucosal infections [174,175,176,177,178]. Whether or not IFN-λ induction in epithelial cells is impaired in aging remains to be determined. In the respiratory and nasal epithelial linings, IFN-λs were the predominant IFN produced early during RSV and IAV infection, and they could confer protection against intestinal RV infection [175,179,180]. Pre-treatment with IFN-λ successfully protected against intravaginal HSV-2 infection in mice, but not non-mucosal LCMV or ECMV infections [181]. Galani et al. demonstrated that IFN-λs act first to defend against low titers of IAV infection and conferred antiviral protection without eliciting broad immune activation, and, as a result, effectively controlled IAV replication in mice without exacerbating immune-mediated lung pathology [182]. Thus, the primary role of type III IFNs during viral infection is to exert antiviral effects to control viral replication, and, as a result, prevent a synergy between heightened viral replication, inflammation, and pathology. While the relationship between aging, viral infection and type III IFN requires further investigation, it can be inferred that impairments on type III IFN production would impair control of viral infection and consequent disease outcome.

## 9. The Therapeutic Potential of IFNs in the Treatment of Inflammation and Infection

There have been many efforts in administering IFNs as treatments for inflammatory diseases, including both autoimmunity and viral infections. However, the required precise timing and magnitude of type I IFNs that distinguish its protective versus pathological inflammatory responses result in difficulties addressing its use as a treatment towards severe viral infections. Recently, COVID-19 clinical trials have outlined that only early administration of type I IFNs can prevent severe disease outcomes [17]. In mouse studies, it is clear that suppressing early viral replication alone through the administration of recombinant IFN-λ is not only effective, but safer than administration of type I IFNs to prevent mortality [182,183]. IFN-λ was also shown to safely and effectively accelerate viral clearance to SARS-CoV-2 infection [184]. Thus, the most effective IFN-based treatments may involve controlling viral replication in order to prevent the exacerbation of inflammatory responses and immunopathology. However, how to therapeutically modulate the IFN response at later stages of infection to safely reduce an already exacerbated immunopathological response requires further study.

Both IFN-γ treatments and adoptive NK cell therapy have also been considered as therapeutics for severe viral infections. In mouse models, IFN-γ administration after infection was capable of improving survival from IAV and EBOV infections [185,186]. NK cell therapies are currently being tested for their ability to control severe SARS-CoV-2 infection [187,188,189]. Type II IFN therapies would have a combined impact through providing antiviral protection, inducing immune responses, and preventing type II mediated immunopathology [12,167]. Contradicting findings have been reported, as other studies found that the inhibition of IFN-γ protected against infection-induced immunopathology [164,190]. Furthermore, NK cell-mediated pathologies during viral infection have also been described, and thus their use in infection is hindered by a need for more research on their regulation during infection [191].

## 10. Discussion

As a consequence of age, as well as comorbidities associated with age, impaired IFN production at early stages of infection leave elderly populations particularly susceptible to viral infections by impairing early viral clearance and dysregulating the immune response. These include an inability to mount a protective immune response regulated by monocyte/macrophages, including NK cell, T cell, and B cell activation, as well as enhanced inflammation by IM and neutrophils, and the impaired resolution of inflammation by macrophages (Figure 2). IFN therapy provides a promising treatment to improve outcomes, but caution must be taken towards their use as therapeutics. While early administration of both type I and type III IFNs may improve viral clearance, delayed administration of type I IFN can exacerbate immune-mediated pathology. As such, their roles at various stages of infection must be further assessed. Furthermore, while delayed and reduced IFN production with aging is evident in elderly populations, other key factors attributed to aging, such as obesity, have correlated with impaired IFN production and warrant further research into their role in infection susceptibility [192].

While this review has focused on the impacts of IFNs during aging, additional questions to consider include how the IFN response to viral infection may influence disease outcomes at the opposite side of the spectrum, in very young populations. In certain diseases, such as SARS-CoV-2, levels of infection remain atypically low in adolescents and children compared to young and middle-aged adults. Thus, whether their improved outcomes correlate with a superior early antiviral response may be of interest for further investigation. At the neonate stage, reduced pDC and type I IFN responses drove pathology during respiratory syncytial infection, a virus with severe outcomes in that demographic [193]. Therefore, an important avenue for the future will be to assess how our IFN responses are shaped by age and other factors when considering susceptibility towards infections. Overall, a better understanding of how type I IFNs determine our responses to viral infection will help up proactively treat viral infections in vulnerable populations.

## Figures and Tables

**Table 1:** Comparison of changes in innate immune responses in aging and the absence of type I IFN.

Cell Type	Aging	Absence/Reduction in Type I IFN
**Neutrophils**	- ↑ neutrophil influx through ↑ IL-17, CXCL1, CXCL2 [61,62]	- ↑ neutrophil recruitment by ↑ CXCL1 and CXCL2 production by monocytes [11,63,64]
**Monocyte/Macrophages**	- ↑ IL-6 and TNF production [30,65] - ↓ macrophage phagocytosis of apoptotic neutrophils [66]	- ↓ IM recruitment [11,27,41,67,68] - ↑ resident IM proliferation [69] - ↓ IM iNOS, ↑ Ly6C^lo^ monocyte iNOS production [63] - ↓ IM TRAIL expression [70,71] - ↓ macrophage phagocytosis and efferocytosis of apoptotic neutrophils [72,73,74,75]
**NK cells**	- ↓ NK cell activation IFN-γ production [76,77] - ↑ NK cell apoptosis [78]	- ↓ NK cell activation and IFN-γ production [27,38,79,80,81] - ↓ NK cell survival [82,83]

**Table 2:** Comparison of changes in adaptive immunity in aging and the absence of type I IFN.

Cell Type	Aging	Absence/Reduction in Type I IFN
**DCs**	- ↓cDC maturation and priming of T cells [109,110,111,112] - ↑ PD-L1/2 on cDCs [111,113]	- ↓ moDC maturation and IL-12 production [114,115,116] - ↓ cDC maturation and priming of T cells [100,117,118,119,120]
**T cells**	- ↓ CD4 and CD8 T cell expansion/survival [110,121,122,123] - ↓ CD8 T cell activation [111,121,122,123,124] - ↓ Sensitivity to IFN-I signalling [125] - ↓ CD4+ Memory T cells [56,105] - ↓ Memory T cell contraction [126] - ↑ PD-1 expression [113]	- ↓ CD8 and CD4 T cell expansion/survival [38,127,128,129] - ↓ CD8 T cell maturation and activation [100,130,131] - ↓ CD8 Memory T cell formation [79,128] - ↓ CD8 Memory T cell cytotoxic function [132] - ↓ Memory T cell contraction [133,134] - ↑ PD-1 expression and exhaustion of CD8+ T cells [127]
**B cells**	- ↓ Vaccine seroconversion [103,135] - ↓ B cell proliferation [136] - ↓ T-bet expression [137] - ↓ Isotype switching [138] - ↓ Affinity maturation [138]	- ↓ B cell proliferation [106,139,140,141] - ↓ Plasma cell differentiation and antibody secretion [106,107,108,142] - ↓ T-bet expression [143,144] - ↓ Isotype Switching [142]

## Data Availability

No new data were created or analyzed in this study. Data sharing is not applicable in this article.
